# Regional Endothermy in a Coral Reef Fish?

**DOI:** 10.1371/journal.pone.0033187

**Published:** 2012-03-05

**Authors:** Justin Q. Welsh, David R. Bellwood

**Affiliations:** Australian Research Council Centre of Excellence for Coral Reef Studies, and School of Marine and Tropical Biology, James Cook University, Townsville, Australia; Institute of Marine Research, Norway

## Abstract

Although a few pelagic species exhibit regional endothermy, most fish are regarded as ectotherms. However, we document significant regional endothermy in a benthic reef fish. Individual steephead parrotfish, *Chlorurus microrhinos* (Labridae, formerly Scaridae) were tagged and their internal temperatures were monitored for a 24 h period using active acoustic telemetry. At night, on the reef, *C. microrhinos* were found to maintain a consistent average peritoneal cavity temperature 0.16±0.005°C (SE) warmer than ambient. Diurnal internal temperatures were highly variable for individuals monitored on the reef, while in tank-based trials, peritoneal cavity temperatures tracked environmental temperatures. The mechanisms responsible for a departure of the peritoneal cavity temperature from environmental temperature occurred in *C. microrhinos* are not yet understood. However, the diet and behavior of the species suggests that heat in the peritoneal cavity may result primarily from endogenous thermogenesis coupled with physiological heat retention mechanisms. The presence of limited endothermy in *C. microrhinos* indicates that a degree of uncertainty may exist in the manner that reef fish respond to their thermal environment. At the very least, they do not always appear to respond to environmental temperatures as neutral thermal vessels and do display limited, but significant, visceral warming.

## Introduction

Marine fishes are largely regarded as ectotherms, incapable of metabolic thermoregulation, and therefore have body temperatures similar to the ambient water temperature [1]–[2]. The optimal body temperatures of organisms, however, often occur within a narrow thermal range depending on the geographic location where they live [3]–[5]. Tropical marine species are usually regarded as being stenothermal, i.e., adapted to a relatively narrow thermal range. Therefore, these tropical stenotherms may have little capacity to deal with large-scale climactic shifts when compared to temperate species [5]–[7].

In terrestrial environments, lizards and other ectotherms have been observed to behaviorally regulate internal temperatures and maintain their bodies within a narrow thermal range [8]–[9]. Like their terrestrial counterparts, several temperate ectothermic fish species are also known to exhibit behavioral thermoregulation. Salmon, for example, are known to actively select cool microenvironments within migratory paths to reduce metabolic processes and conserve energy [10]. In aquarium-based trials, the reef fish species *Zebrasoma flavescens* [11], *Balistes fuscus*, *B. vidua*, *Canthigaster jactator*, *Cromileptes altivelis*, *Forcipiger longirostris* and *Naso lituratus* [12] have all displayed the ability to behaviorally thermoregulate, actively seeking out relatively stable ambient temperatures.

In contrast, a few fish species exhibit endothermy. In teleost endotherms, using physiological mechanisms, such as intricate counter-current blood flow systems, they are able to retain locally produced metabolic heat, thereby increasing internal temperatures [13]. In this way, lamnid sharks, tunas, other scombrids and billfishes are able to use regional endothermy to heat key body regions, maintaining optimal metabolic functioning when feeding in cool environments [14]–[16]. Such thermoregulation has only been documented in pelagic predators. For benthic fishes, behavioral thermoregulation is the only documented mode of regulating body temperatures, with little field-based work conducted on the thermal biology of reef fish.

Parrotfishes (Labridae, formerly Scaridae) are found throughout tropical and temperate reef habitats where they play a critical role in ecosystem processes on coral reef systems [17]–[18]. As important reef herbivores and bioeroders, parrotfishes have become a central focus of research on coral reef resilience when dealing with global climate change, habitat degradation, and other anthropogenic factors threatening reefs [19]–[20]. On Pacific reefs one species stands out, *Chlorurus microrhinos*, the steephead parrotfish. Abundant and large, this parrotfish species is particularly important, contributing heavily to the ecosystem processes of bioerosion and algal removal [21]–[23]. *C. microrhinos* is a model example of a functionally important reef fish and one in which little is know about their thermal biology.

In this study, our aim was to assess the potential for regional endothermy in *C. microrhinos* by monitoring individuals' internal temperature relative to their ambient temperatures. More specifically, the internal temperature of *C. microrhinos* relative to that of its environment was evaluated over a full 24 h period to determine the nature and extent of thermoregulation on the reef and in aquaria. The basic question was: are parrotfish capable of regional endothermy in the wild?

## Materials and Methods

### Study site, collection and tagging

This study was conducted between April and June 2010 on Orpheus Island, Queensland, Australia (18°35′S, 146°20′E), on the fringing reef of Pioneer Bay on the leeward side of the island. Adult *C. microrhinos* were captured from the reef crest using monofilament barrier nets (50×2 m, 35 mm square). Description of the study area is provided in Fox and Bellwood [23].

Individual *C. microrhinos* were anaesthetized in a tricane methanesulfonate (MS-222) seawater solution (0.13 gL^−1^) for approximately 60 s, until loss of equilibrium. At this time, the fork length (FL; cm) was recorded and an ultrasonic transmitter (tag; V9T-2H, Vemco) was inserted into the peritoneal cavity of each individual through a small incision made at the mid-point between the pectoral fin base and the anus. Once tagged, incisions were treated with an antiseptic and closed using nylon sutures (Ethelon). All the methods utilized in the present study were approved by James Cook University Animal Ethics Committee (A 1321) and under the permit requirements for the Great Barrier Reef Marine Park Authority, permit number: G08/28894.1.

### Sampling protocol

Tagged individuals were given a 24 h recovery period in a large 3,300 L (2460 mm diameter×700 mm height) flow-through tank, to minimize tagging effects and to give the incision time to heal. After 24 hrs, fish were inspected for any signs of infection. No infection was observed and all individuals displayed normal behavior.

The temperature of the peritoneal cavity of each *C. microrhinos* in captivity and on the reef was obtained at 15 min intervals. Sampling occurred over a 24 h period to encapsulate a full diurnal environmental thermal regime. Data were obtained from the internal tag, using a directional hydrophone (VH 110, Vemco) and receiver unit (VR100, Vemco). Environmental thermal data were collected from within the tank and on the reef using thermal data loggers (HOBO Water Temp Pro v2).

Before thermal data from the environmental sensors and tags were used, all were calibrated against each other. Because of the small temperature ranges anticipated, extreme care was taken with cross calibration. Tags (V9T-2H, Vemco) and thermal data loggers (HOBO Water Temp Pro v2) were placed in a thermally homogeneous aquarium with mild water flow, uniform light exposure, and no bubbles on or around sensors. Each tag and sensor was then left to record the water temperature for at least 5 h. Pilot studies found no variation as a result of different calibration temperatures, however, short calibration periods (<15 min) were found to lack the required precision. After 5 h, results were compared and a calibration coefficient was generated for each tag or sensor, which was used to calibrate all subsequent thermal readings. This calibration was applied to the data and accounted for the small thermal variation (<0.09°C), which may arise due to differences in two different sensors used to record thermal readings.

In order to obtain thermal profiles from fishes in a relatively stable thermal environment, three individual *C. microrhinos* were placed separately in large 3,300 L (2460 mm diameter×700 mm height) flow-through tanks. After the 24 h recovery period, temperatures were recorded over a full 24 h period for each individual.

To obtain thermal profiles from fish on the reef, a total of 5 tagged individual *C. microrhinos* were released into Pioneer Bay at the site of capture. Fish were then located using a 3.1 m kayak fitted with a directional hydrophone (VH110, Vemco) during daylight hours and from a 4.6 m aluminum dinghy during the night. Individuals were tracked for a 24 h cycle, commencing at 06:00 hrs when they were located in their respective sleeping sites on the reef base, approximately one hour before the fish moved onto the reef. Thermal and positional data was recorded for each individual as they followed normal movement patterns throughout their home ranges [24].

Environmental temperatures were recorded on the outer reef flat (−0.3 m; depth values reported below chart datum), the reef crest (0.5 m), the subtidal reef crest (1.5 m), the reef slope (4.8 m), and the reef base (6.4 m) using thermal data loggers. Thermal data loggers were placed within the home range of the tagged individuals and were distributed according to the habitat utilization of *C. microrhinos*. Environmental readings were taken at 15 min intervals, which correspond to the timing at which thermal data was collected from the fish. Location data from each individual was plotted in ArcGIS 9.0 to determine which of the thermal data loggers corresponded to the position of each *C. microrhinos* at the time when the temperature readings were taken. The thermal loggers were therefore in the same reef zone and within 15 m of the fish at each data recording. The majority of records were within 5 to 10 m. At night, however, thermal loggers were placed within 1 to 3 m of the known resting sites to ensure accurate environmental readings without disturbing the fish.

The extent of within-habitat thermal variation also had to be evaluated to ensure that the 3 m proximity to fishes sleeping sites was sufficient to accurately represent the temperature within the fish's sleeping sites. To do this, following the main experiment, data loggers were placed in *C. microrhinos* sleeping sites (one inside the sleeping hole, one outside and one within 5 m). Sleeping sites were confirmed by the presence of an intact mucous cocoon or a visual sighting of a sleeping *C. microrhinos*. Thermal variation was examined at 6 sleeping sites. At each site, data were recorded for a full 24 h period.

### Data analysis

To evaluate thermal differences between the peritoneal cavity of *C. microrhinos* and its environment, the peritoneal cavity temperature readings were subtracted from the immediate environmental temperature readings to yield a thermal difference value, to be used in subsequent analysis:
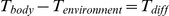



Diurnal sampling periods were defined as being between 06:30 hrs when fish became active and 17:45 hrs when fish returned to sleeping sites. Nocturnal sampling periods were defined as the sampling time between 18:00 hrs and 06:15 hrs when fish remained stationary in their sleeping sites. The amount of variability in reef-based diurnal and nocturnal samples was assessed using a coefficient of variance (CV; standard deviation/mean) calculated on the average *T_diff_* values for each sampling period (diurnal and nocturnal) in tanks and on the reef. To evaluate whether the average internal temperature difference (*T_diff_*) of individuals on the reef (n = 5) and in aquaria (n = 3) during diurnal and nocturnal samples differed from zero, a t-test for single means was used. The test was justified as the t-test is appropriate for small samples. A log_10_ transformation was applied to the nocturnal sample from the reef to meet the assumption of normality in the data.

## Results

**Figure 1:**
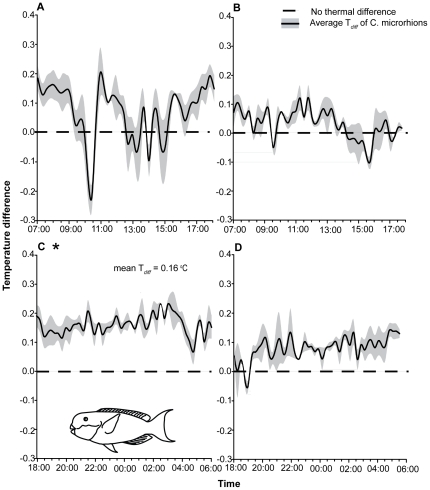
Temperature difference profiles of *Chlorurus microrhinos* over a 24-hour period. Mean *T_diff_* values recorded (°C above or below ambient ± SE) in *C. microrhinos* **A.** on the reef during the day while fish are active (07:00–15:45 hrs; n = 5), **B.** in tanks during the day (07:00–15:45 hrs; n = 3), **C.** on the reef at night while individuals are in sleeping sites (18:00–06:00 hrs; n = 5) and **D.** in tanks during the night (08:00–06:00 hrs; n = 3). * Indicates significance at 0.01.

The internal temperature of *C. microrhinos* held in tanks remained near ambient temperatures (*T_diff_* diurnal = 0.04±0.02°C; mean ± SE; *T_diff_* nocturnal = 0.08±0.03°C; Figure 1b, d) and consistently tracked environmental conditions with low variability (CV diurnal = 0.63; CV nocturnal = 0.64) (Figure S2). However, reef-based fish behaved quite differently. At night, fish on the reef were consistently and significantly warmer than the environment (Figure S1). The average nocturnal *T_diff_* value from individuals on the reef was 0.16±0.01°C, with low variability (CV = 0.50) and was significantly different from zero (*T* = 13.04, *P* = 0.0002; Figure 1c). Diurnal samples on the reef did not follow similar trends, with peritoneal cavity temperatures found to be, on average, 0.08±0.01°C above ambient, and quite variable (CV = 3.16). Furthermore, the mean diurnal temperature differences did not differ significantly from zero (*T* = 2.10, *P*>0.05; Figure 1a).

The precision of the tags used in the present study to monitor individuals' visceral temperature is not high when dealing with the relatively small thermal excess detected in the present study. However, the consistency at which all five fish demonstrate a similar pattern of visceral warming on the reefs cannot be ignored. Indeed, the limited precision of the tags should hinder our capacity to detect a difference between the internal temperature of captive and wild individuals (as well as nocturnal and diurnal periods). The fact that a significant difference was detected despite the potential for tag error emphasizes the fact that there is a significant discrepancy between the internal temperature of fish on the reef at night and their environment, and that this pattern is not an artifact of tag error.

Microhabitat temperature variability on the reef-base in the vicinity of *C. microrhinos* sleeping sites was low. The temperature within sleep sites was consistently cooler (−0.07±0.004°C) than the sites immediately outside and within 5 m of sleeping holes (Figure S3). This suggests that thermal difference values for the peritoneal cavity of *C. microrhinos* relative to the environment are conservative.

## Discussion

In this study we demonstrate a degree of regional endothermy in *C. microrhinos* in the field with no such pattern observed in fish held in tanks. Behavioral thermoregulation is well documented in reef fishes [11]–[12] but endothermy is highly unusual. Muscular temperatures 1 to 2°C above ambient have been reported in many teleost fishes and is attributed to heat generated from muscle activity, which is rapidly lost from the body due to the physical properties of water [25]–[26]. *C. microrhinos* measured in this study exhibited no increase in temperature during the day when muscular activity was occurring. At night fish were completely stationary and thermal readings were taken from areas with minimal musculature. We cannot attribute the elevated temperature to simple muscle-generated heat. This raises the question; how is it achieved?

Consistent elevated temperatures in the peritoneal cavity of *C. microrhinos* at night, while the fish are stationary, indicates that this species possesses non-muscular mechanisms that maintain an elevated peritoneal cavity temperature. This is not homeostasis and even ‘thermoregulation’ appears to be limited. Rather, it appears to be heat production with a limited capacity to raise internal temperatures slightly above ambient temperatures. We cannot discount behavioral thermoregulation during the day, but this study does demonstrate a degree of limited regional endothermy in a reef fish species at night. The mechanisms by which regional endothermy is occurring are not yet clear, however, physiological and dietary attributes of *C. microrhinos* offers two alternative explanations for heat production: exogenous or endogenous sources.

Exogenous heat within the peritoneal cavity of individuals may result from fermentation activity within the guts. In sheep, fermentation activities in the rumen result in an elevation of portal vein blood temperatures by 0.07°C, which was attributed to heat production from microbial fermentation [27]. Although microbial symbionts have been described from the guts of many herbivorous reef fishes [28]–[31], *C. microrhinos* have very low concentrations of short-chain fatty acids in their hindgut, indicating limited bacterial fermentation [32]–[34]. The potential contribution of fermentation is further reduced by their gut physiology, as *C. microrhinos* have a short gut and they empty the vast majority of the gut contents before entering a sleeping site at night [22], [32]. It would therefore be expected that heat production would decrease throughout the night as what little substrate for fermentation remained in the gut became depleted, rather than exhibiting a consistently elevated internal temperature as observed. Overall, it is highly unlikely that the elevated thermal environment of the peritoneal cavity of *C. microrhinos* is a result of fermentation.

Despite metabolic heat production and retention being widespread in endothermic species, to date, only brown adipose tissue in mammals [35] and specialized heat producing skeletal muscle cells in billfishes [36] have been identified as functioning exclusively for thermogenesis. Though they do not utilize dedicated tissues for thermogenesis, lamnid sharks and tunas retain heat generated from red muscle activity within their cores [16], [37]. The relatively small elevation in peritoneal temperature of *C. microrhinos* suggests that the presence of dedicated tissues is unlikely. Furthermore, as significant endothermy was only recorded at night when fish are stationary, the slightly elevated peritoneal cavity temperature in *C. microrhinos* does not appear to originate from locomotory muscle contractions. Instead, it is more likely that the heat production within the peritoneal cavity may arise from the metabolic activity of the digestive processes operating at night.

In mammals, the metabolic rate of the liver increases dramatically after a protein rich meal, as protein synthesis commences within the liver [3]. The large liver of *C. microrhinos* relative to its body size (2.93% of total body weight compared to 0.67–2.17% in other reef fishes; Table S1) and the primary dietary constituents of this species being protein [33], suggests that an increase in metabolic activities in the liver at night may be generating a significant metabolic heat. This is supported by the minimal elevation of internal temperatures detected when fish were held in aquaria at night, as they were stationary while sleeping (similar to the reef based individuals) and not able to feed the day prior to data collection and thus no new protein substrates would be available for processing within the liver.

For gastro-intestinal metabolism to elevate the internal thermal environment, any heat generated must be locally retained. Physiological mechanisms have evolved in all fish lineages that exhibit regional endothermy, to reduce the loss of metabolic heat [16], [38]. Vascular counter-current heat exchange systems have arisen to retain metabolically generated heat in the brain and eyes of billfishes and in the red muscle blocks of tunas [16], [36], [39]. It is possible that a simplified version of such counter-current heat exchange may exist in the vascular systems supplying the peritoneal cavity of *C. microrhinos*, resulting in localized retention of heat generated from digestive processes. Alternatively, a relatively basic counter-current heat exchange mechanism may already exist in the vascular system of fishes. The parallel arrangement of the arteries and veins may act as a site for heat transfer, retaining some visceral heat which would otherwise be lost from venous blood as it moves towards the gills [40]–[41].

In the marine environment, heat rapidly dissipates into the surrounding water [38]. The gills are responsible for 80 to 90% of metabolic heat lost in fishes while they are active, reflecting the high rates of blood flow [42]–[43]. The ease by which heat is lost through the gills may account for the high variability in the diurnal internal temperatures of *C. microrhinos* as individuals move through the water column. However, at night, when stationary, blood flow to the gills may be restricted, reducing the total metabolic heat lost during respiration [43]. Furthermore, vasoconstriction in peripheral systems, as seen in marine mammals [38], may reduce heat loss at night via the fins and body. Passive mechanisms may also be involved including a black-silver lining of the peritoneal cavity (cf. vacuum flasks) and the mucous cocoon. Acting as a barrier to extensive water movement, water within the cocoon may become slightly warmer than that of the surrounding environment, reducing the rate of convective heat loss. These mechanisms, however, require further investigation.

Some ectotherms such as marine iguanas utilize mass specific heat loss to exploit colder microhabitats for foraging and shelter [44]–[45]. As such, they are able to remain in thermally unfavorable environments as heat slowly dissipates from within their body. The absence of a steady decline in temperature in *C. microrhinos* at night suggests that the limited endothermy within the peritoneal cavity is not occurring as a result from heat retained from diurnal foraging in warmer, shallower waters.

Overall, when on the reef at night, *C. microrhinos* produces heat, which is locally retained and results in consistently elevated internal temperatures. In tanks or during the day on reefs, however, internal temperatures do not significantly depart from ambient. All evidence points to endogenous heat from the viscera paired with some heat retention mechanism. However, the function or potential benefits of visceral warming in *C. microrhinos* remains to be determined.

## Supporting Information

Figure S1 **Thermal profile of 5 individual** ***Chlorurus microrhinos*** **sampled on the reef and the corresponding environmental temperatures at 15 minute sampling intervals.**
(TIF)

Figure S2 **Thermal profile 3 individual** ***Chlorurus microrhinos*** **held in tanks and the corresponding tank temperatures at 15 minute sampling intervals.**
(TIF)

Figure S3 **Assessment of the thermal microhabitat variability on the reef base between six parrotfish sleep sites, the area immediately outside sleeping sites and 5 m away from sleep sites over a 24 hour period.**
(TIF)

Table S1 **Mean relative liver weights of coral reef fishes (from Bellwood 1985).**
(DOC)
