# Supplementary figures and images for: Regional Endothermy in a Coral Reef Fish?
Source: PLoS One. 2012 Mar 5;7(3):e33187. doi: 10.1371/journal.pone.0033187 (PMC3293926; doi:10.1371/journal.pone.0033187)

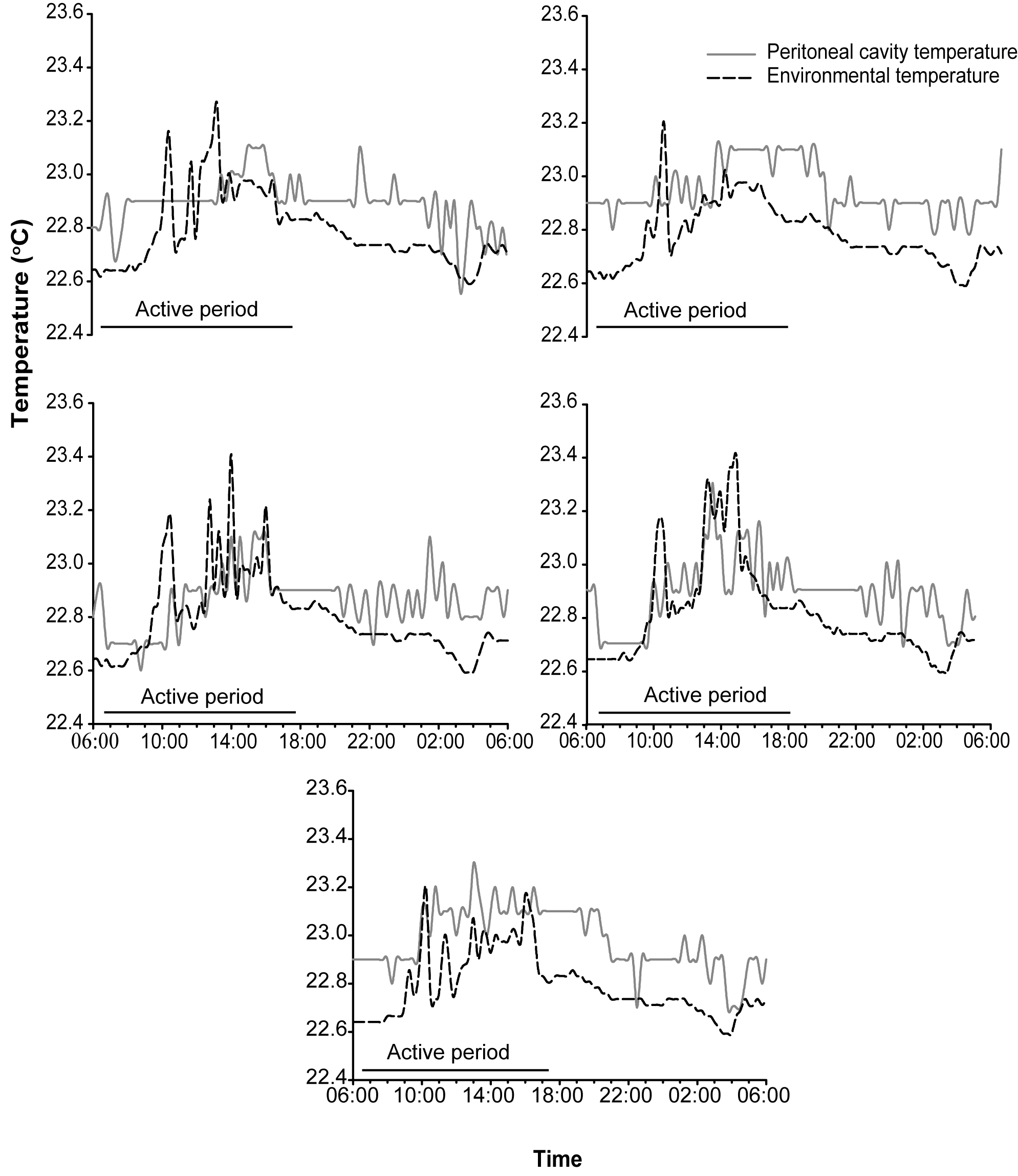

Supplement: Figure S1 — Thermal profile of 5 individual Chlorurus microrhinos sampled on the reef and the corresponding environmental temperatures at 15 minute sampling intervals. (TIF) [file pone.0033187.s001.tif]

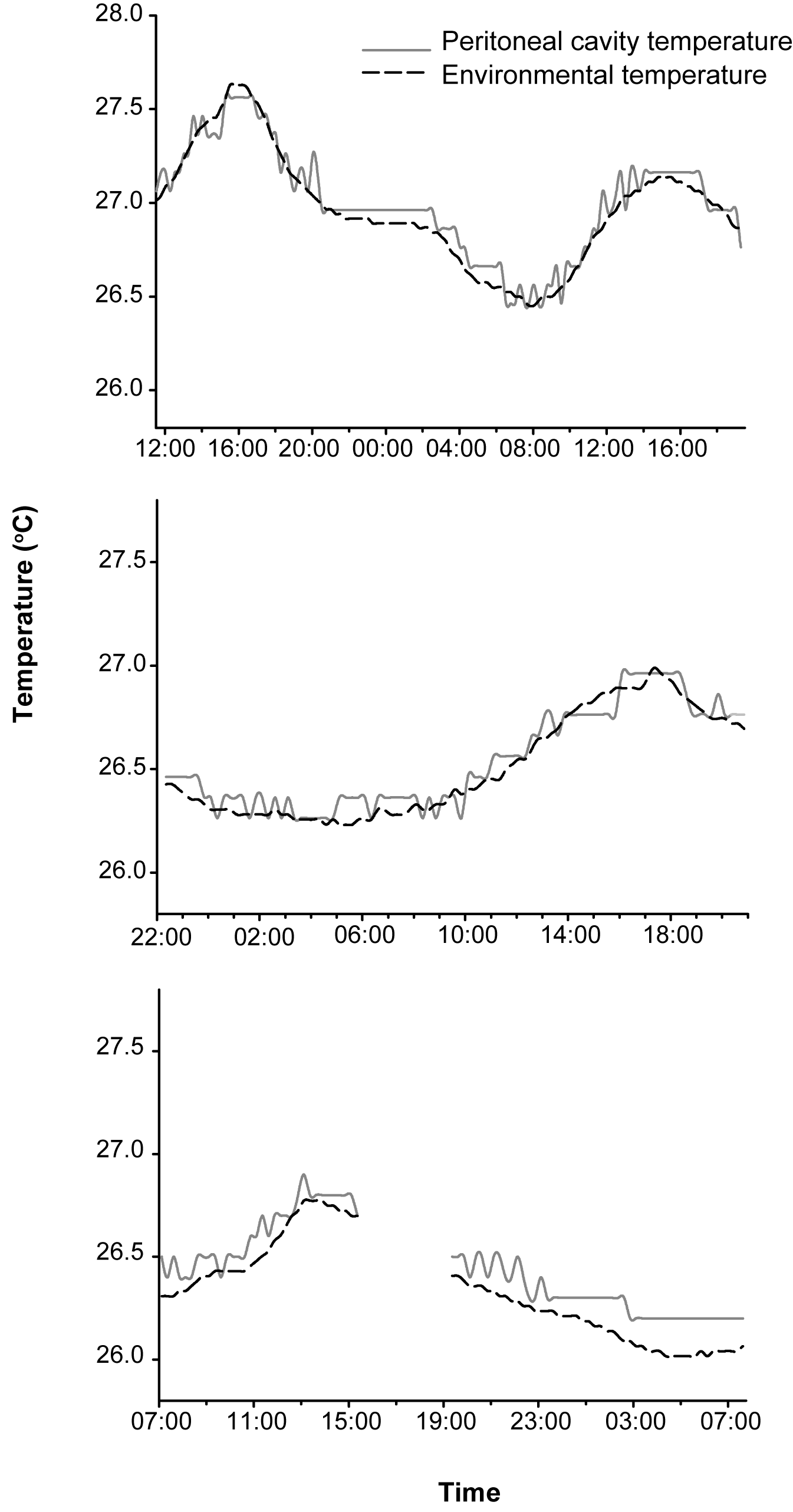

Supplement: Figure S2 — Thermal profile 3 individual Chlorurus microrhinos held in tanks and the corresponding tank temperatures at 15 minute sampling intervals. (TIF) [file pone.0033187.s002.tif]

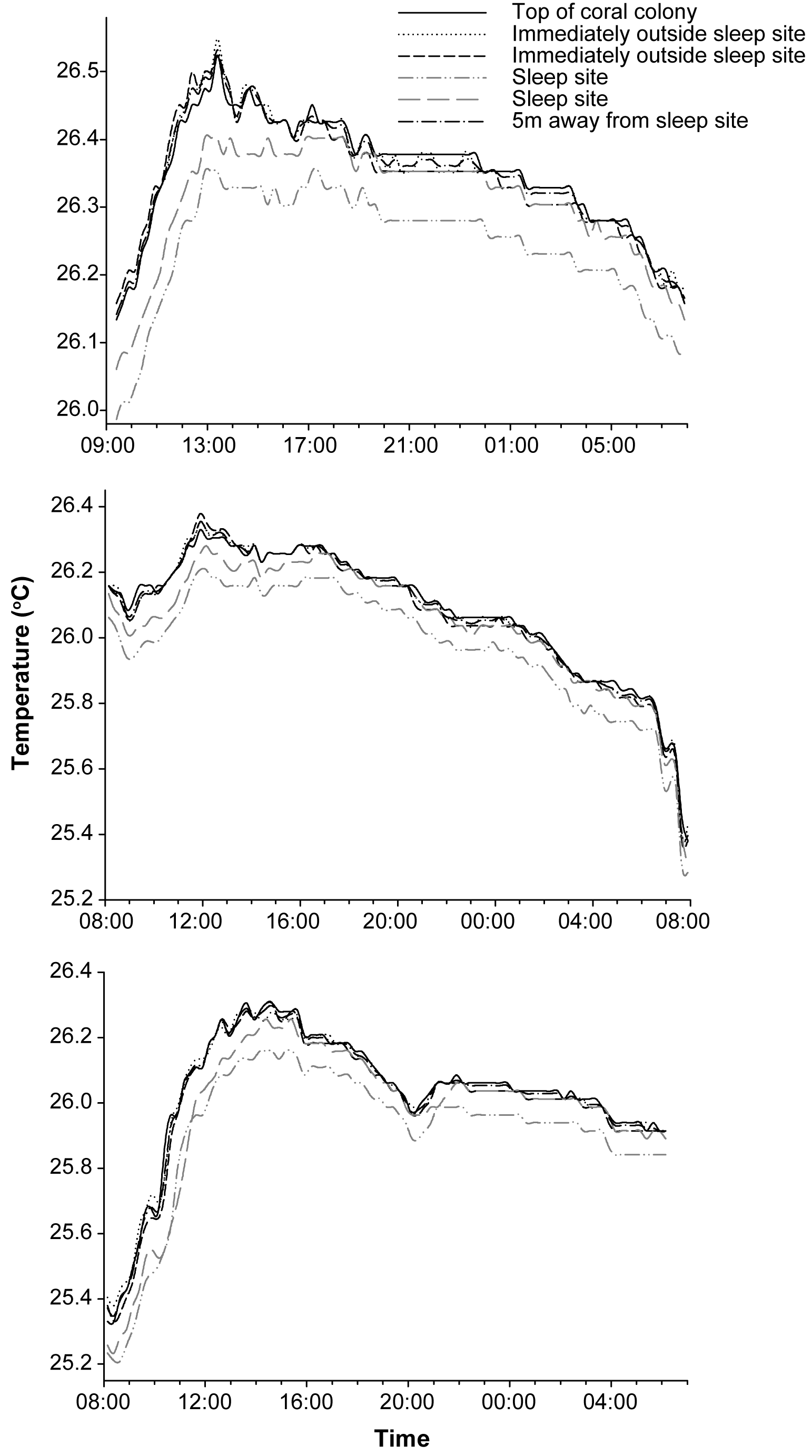

Supplement: Figure S3 — Assessment of the thermal microhabitat variability on the reef base between six parrotfish sleep sites, the area immediately outside sleeping sites and 5 m away from sleep sites over a 24 hour period. (TIF) [file pone.0033187.s003.tif]
