# Supplementary material for: Regional Endothermy in a Coral Reef Fish?
Source: PLoS One. 2012 Mar 5;7(3):e33187. doi: 10.1371/journal.pone.0033187 (PMC3293926; doi:10.1371/journal.pone.0033187)
Supplement: Table S1 — Mean relative liver weights of coral reef fishes (from Bellwood 1985). (DOC) [file pone.0033187.s004.doc]

| **Supplemental table 1.** Mean relative liver weights of coral reef fishes (from Bellwood 1985) | | | |
| --- | --- | --- | --- |
| **Species** | **Number** | **Standard Length range (mm)** | **Liver weight (% body mass)** |
| **Scarids:** |  |  |  |
| *Scarus rivulatus* | 1 | 280 | 4.13 |
| *Chlorurus microrhinos* | 1 | 370 | 3.10 |
| *Chlorurus sordidus* | 1 | 310 | 2.93 |
|  |  |  |  |
| **Non-scarid herbivores:** |  |  |  |
| *Naso unicornis* | 2 | 235-370 | 1.83 |
| *Naso tuberosus* | 1 | 345 | 2.17 |
| *Siganus lineatus* | 2 | 355 | 1.20 |
| *Siganus spinus* | 1 | 290 | 1.80 |
| *Acanthurus dussumieri* | 1 | 285 | 0.67 |
|  |  |  |  |
| **Carnivores/omnivores** |  |  |  |
| *Lutjanus carponotatus* | 4 | 280-300 | 0.76 |
| *Plectrorhynchus flavimaculatus* | 1 | 380 | 1.20 |
| *Spilotichtys pictus* | 1 | 410 | 0.78 |
| *Lethrinus* sp. | 2 | 305-320 | 1.14 |
| *Epinephelus flavocaeruleus* | 1 | 370 | 0.67 |
| *Plectropomus leopardus* | 1 | 325 | 0.91 |
| *Epinephelus merra* | 6 | 240-330 | 0.82 |
| *E. fasciatus* | 4 | 260-330 | 0.56 |
| *Choerodon schoenleinii* | 1 | 235 | 2.44 |
| *Chaetodon lineolatus* | 1 | 160 | 1.41 |
| *Pomacanthus semicirculatus* | 1 | 270 | 0.90 |
| *Sargocentron spiniferum* | 2 | 310 | 1.00 |
| *Caesio cuning* | 1 | 280 | 1.10 |

**Supplemental References**

Bellwood DR (1985) The functional morphology, systematics and behavioural ecology of parrotfishes (family Scaridae). PhD dissertation, James Cook University, Townsville, Queensland.

Available online at: http://eprints.jcu.edu.au/2052/
